# Learning from Ebola: readiness for outbreaks and emergencies

**DOI:** 10.2471/BLT.15.165720

**Published:** 2015-12-01

**Authors:** Margaret Chan

**Affiliations:** a World Health Organization, avenue Appia 20, 1211 Geneva 27, Switzerland.

For almost 70 years, the World Health Organization (WHO) has coordinated the norms and technical standards required to improve global health. This is the role people most often associate with WHO. However, the organization’s constitution also calls on it to “furnish technical assistance and, in emergencies, necessary aid” to governments, a role WHO has played on countless occasions. Examples include eradicating smallpox, bringing polio and guinea-worm disease to the brink of eradication and responding to health threats posed by humanitarian crises worldwide.

Despite initial delays in the western Africa Ebola outbreak response, the tide of this unprecedented health crisis has now been turned. While still requiring intense and focused action to bring new cases to zero, the outbreak is now limited to only a few cases per week. Deficiencies in capacity, expertise and approach revealed by WHO’s response to Ebola suggest that organization-wide change is needed:^1^ WHO must ensure it can prepare for and respond to outbreaks and emergencies in a way that genuinely supports national efforts and fully integrates with international partners.

WHO must do more than provide support in emergencies. It must become a fully operational emergency organization. This distinction may sound subtle – but the new course that WHO is charting marks one of the most profound shifts in its history. WHO must enable countries to strengthen their outbreak and emergency preparedness, while ensuring that its own experts and those of its partners can rapidly roll out the required response within the first 24–72 hours. Subsequently, WHO must support countries in the recovery phase after an outbreak or emergency and help them “build back better” when health systems have been damaged. To succeed, this will require stronger, more functional linkages and partnerships to allow WHO to prepare and respond more predictably to infectious disease outbreaks and other emergencies.

WHO has established a high-level advisory group,^2^ chaired by Dr David Nabarro, the United Nations Secretary-General’s Special Envoy for Ebola. The advisory group is mandated to provide WHO with clear, actionable advice on developing the capacities needed to coordinate governments, national responders, United Nations (UN) agencies, funds and programmes and operational partners in outbreaks and emergencies.

The Ebola outbreak has taught us many lessons, among them that the response to outbreaks and emergencies must start and end at ground level – which means that certain key capacities have to be in place before launching a response, including leadership and coordination, technical support, logistics, management of human resources and communications. It has also shown that the organizations working to contain outbreaks and emergencies must collaborate closely. Following the 68th World Health Assembly decision on the Ebola virus disease outbreak and the Special Session of the Executive Board on Ebola,^3^^,^^4^ WHO has begun reviewing systems and capacities throughout the organization to streamline the way it works in outbreaks and emergencies.

These changes focus on six key areas:^5^ (i) a unified WHO platform for outbreaks and emergencies with health and humanitarian consequences; (ii) a global health emergency workforce, to be effectively deployed in support of countries; (iii) core capacities at country-level under the International Health Regulations; (iv) functioning, transparency, effectiveness and efficiency of the International Health Regulations; (v) a framework for research and development preparedness and capacity during outbreaks or emergencies; and (vi) adequate international financing for pandemics and other health emergencies, including a 100 million United States dollars contingency fund and a pandemic emergency financing facility.

By the end of 2015, a structure will be finalized for a new operations platform, which will lead to a major transformation of WHO. The platform will improve and align the strengths and capacities of the country, regional and headquarters’ levels of the organization into a single team. There will be an emphasis on overcoming managerial, administrative and logistic constraints, improving performance and leveraging strategic partnerships in outbreak and humanitarian emergency preparedness and response.

A clear incident management system will be developed and understood by all inside and outside the organization. For this purpose, WHO has been sounding out all relevant constituencies on the steps it must take. These include operational humanitarian and disease outbreak control partners from global health cluster and Global Outbreak Alert and Response Network partners. Member States are closely following and guiding the process, providing support to ensure that WHO can deliver the services governments need in emergencies.

No single organization can deliver the wide range of services and systems needed for a truly global mechanism that prepares for and responds to outbreaks and emergencies. This is why WHO will continue seeking advice from our partners inside and outside the UN system to make needed change. With their collaboration and support, WHO will be well positioned to deliver what the world needs when outbreaks and emergencies occur: a timely response that rapidly contains the consequences – for economies and societies as well as for human health.
